# Luteolin inhibits GPVI-mediated platelet activation, oxidative stress, and thrombosis

**DOI:** 10.3389/fphar.2023.1255069

**Published:** 2023-10-31

**Authors:** Yujia Ye, Lihong Yang, Min Leng, Qian Wang, Jiankui Wu, Wen Wan, Huawei Wang, Longjun Li, Yunzhu Peng, Shengjie Chai, Zhaohui Meng

**Affiliations:** Laboratory of Molecular Cardiology, Department of Cardiology, The First Affiliated Hospital of Kunming Medical University, Kunming, China

**Keywords:** luteolin, antiplatelet, collagen, GPVI, thrombus formation, platelet production

## Abstract

**Introduction:** Luteolin inhibits platelet activation and thrombus formation, but the mechanisms are unclear. This study investigated the effects of luteolin on GPVI-mediated platelet activation *in vitro* and explored the effect of luteolin on thrombosis, coagulation, and platelet production *in vivo*.

**Methods:** Washed human platelets were used for aggregation, membrane protein expression, ATP, Ca^2+^, and LDH release, platelet adhesion/spreading, and clot retraction experiments. Washed human platelets were used to detect collagen and convulxin-induced reactive oxygen species production and endogenous antioxidant effects. C57BL/6 male mice were used for ferric chloride-induced mesenteric thrombosis, collagen-epinephrine induced acute pulmonary embolism, tail bleeding, coagulation function, and luteolin toxicity experiments. The interaction between luteolin and GPVI was analyzed using solid phase binding assay and surface plasmon resonance (SPR).

**Results:** Luteolin inhibited collagen- and convulxin-mediated platelet aggregation, adhesion, and release. Luteolin inhibited collagen- and convulxin-induced platelet ROS production and increased platelet endogenous antioxidant capacity. Luteolin reduced convulxin-induced activation of ITAM and MAPK signaling molecules. Molecular docking simulation showed that luteolin forms hydrogen bonds with GPVI. The solid phase binding assay showed that luteolin inhibited the interaction between collagen and GPVI. Surface plasmon resonance showed that luteolin bonded GPVI. Luteolin inhibited integrin αIIbβ3-mediated platelet activation. Luteolin inhibited mesenteric artery thrombosis and collagen- adrenergic-induced pulmonary thrombosis in mice. Luteolin decreased oxidative stress *in vivo*. Luteolin did not affect coagulation, hemostasis, or platelet production in mice.

**Discussion:** Luteolin may be an effective and safe antiplatelet agent target for GPVI. A new mechanism (decreased oxidative stress) for the anti-platelet activity of luteolin has been identified.

## 1 Introduction

Cardiovascular diseases are currently the leading cause of premature death and disability worldwide, with ischemic heart disease and stroke being the main culprits (GBD, 2020; Roth et al., 2020). Antiplatelet therapy is an important tool in preventing and treating cardiovascular diseases (Flumignan et al., 2022; Gutierrez et al., 2022; Kamarova et al., 2022). Although antiplatelet drugs effectively reduce the incidence of adverse cardiovascular events, they also increase the risk of bleeding side effects (Jourdi et al., 2021; Fuentes, 2022). Therefore, searching for efficient and safe antiplatelet drugs is important.

Luteolin is the most common flavonoid in many vegetables, fruits, and herbs. Luteolin has antioxidant, antitumor, anti-inflammatory, and cardioprotective activities (Luo et al., 2017; Ashrafizadeh et al., 2020; Slika et al., 2022). Luteolin significantly inhibits collagen, U46619, and ADP-induced activation in human platelets, which may be related to the direct binding of luteolin to T_X_A_2_ receptors (Guerrero et al., 2005; Guerrero et al., 2007; Lu et al., 2021). Choi et al. (2015) found that luteolin effectively inhibits thrombin and coagulation factor X activity and significantly inhibits FeCl_3_-induced carotid thrombosis in mice. Luteolin also possesses protective effects against oxidative stress. It can alleviate H_2_O_2_-induced p38 MAPK and NF-κB activation through a reactive oxygen species (ROS)-dependent pathway (Chen et al., 2020).

At the site of vascular injury, platelets adhere to and aggregate in the exposed endothelial extracellular matrix (ECM) (McFadyen et al., 2018). Among the multiple components of the ECM, collagen is considered a potent platelet agonist; collagen provides an adhesion matrix for the platelets and directly induces significant cellular activation (Fuentes, 2022). The platelet surface expresses several receptors that interact with collagen, among which integrin a2β1 and the IgG-like receptor glycoprotein VI (GPVI) are particularly important (Davì and Patrono, 2007). Integrin a2β1 mainly mediates adhesion, whereas GPVI is central and essential for activating platelet collagen receptors (Borst and Gawaz, 2021). *In vitro* and *in vivo* experiments revealed that a lack of GPVI receptors or blockade of the GPVI receptors effectively inhibits platelet activation and reduces thrombosis and that these interventions are accompanied by only a slight bleeding tendency, probably because GPVI is expressed only on platelets and their precursor megakaryocytes (Lebozec et al., 2017; Martins Lima et al., 2019; Mayer et al., 2021; Uphaus et al., 2022). Therefore, anti-GPVI is increasingly considered a safe and efficient antiplatelet target compared to traditional antiplatelet agents (Martins Lima et al., 2019; Borst and Gawaz, 2021). GPVI and oxidative stress share some relationship (Mondal et al., 2018), and GPVI can generate oxidative stress (Qiao et al., 2018).

Luteolin has significant antiplatelet activity and antithrombotic effects, but whether luteolin affects platelet-GPVI interaction and inhibits platelet activation and oxidative stress in thrombosis has not been reported. Therefore, the present study investigated the effect of luteolin on GPVI-mediated platelet activation *in vitro* and explored the effect of luteolin on thrombosis, coagulation, oxidative stress, and platelet production *in vivo*. This study could provide new refinements on the antiplatelet mechanisms of luteolin.

## 2 Materials and methods

### 2.1 Animals and human platelets

C57BL/6 mice (n = 152, males, 4–6 weeks old, 16–20 g) were purchased from the Department of Experimental Animal Science of Kunming Medical University (Kunming, China). The protocols for the *in vivo* experimental studies in mice were reviewed and approved by the Experimental Animal Ethics Committee of Kunming Medical University (approval # kmmu2021737). Human blood samples were obtained from 10 healthy volunteers from the hospital and research staff who had not taken antiplatelet or non-steroidal anti-inflammatory drugs (NSAIDs) for at least 14 days before blood collection. The study protocol was approved by the Ethics Committee of the First Affiliated Hospital of Kunming Medical University in accordance with the Helsinki Declaration on the Use of Human Subjects (approval # 2020-L-17). All volunteers signed an informed consent form.

### 2.2 Platelet preparation

Blood was collected from a median cubital vein using a 3.8% sodium citrate anticoagulation tube with a 1:9 dilution ratio. The platelets were washed using a previously described method with slight modification (Babur et al., 2020; Uphaus et al., 2022). The blood was centrifuged at room temperature at 300 × g for 10 min to separate platelet-rich plasma (PRP). PRP was added to 50 ng/mL of PGE1 and 5 mM EDTA, centrifuged at 2,000 × *g* for 2 min at room temperature, and the platelet precipitate was obtained after discarding the supernatant. The modified HEPES-Tyrode’s buffer (20 mM 4-(2-Hydroxyethyl)-1-piperazine ethane sulfonic acid [HEPES], pH 7.3, 129 mM NaCl, 2.9 mM KCl, 12 mM NaHCO_3_, 0.34 mM Na_2_HPO_4_, 20 mM HEPES, 5 mM D-Glucose, 1 mM MgCl_2_) was used to resuspend the platelets, added with 50 ng/mL PGE1 and 5 mM EDTA, and the platelets were washed by centrifugation at 2,000 ×*g* for 2 min at room temperature. The washed platelets (WP) were resuspended using modified HEPES-Tyrode’s buffer, and the platelet count was adjusted to 150–200 × 10^9^/L.

### 2.3 Platelet aggregation and ATP release assay

Platelet aggregation and ATP release were detected using the Chrono-Log platelet aggregation instrument (Chrono-Log Corp., Havertown, Pennsylvania, United States), as previously reported (Babur et al., 2020). WPs (200 × 10^9^/L) were co-incubated with luteolin (2.5, 5, 10, 25, and 100 μM; MedChemExpress, Monmouth Junction, NJ, United States) or DMSO for 10 min at 37°C. In addition, 1 mM CaCl_2_ and different platelet activators (ADP, collagen, and thrombin; Chrono-Log Corp., Havertown, Pennsylvania, United States; U46619, from Sigma, St. Louis, MO, United States; convulxin, sc-202554 from Santa Cruz Biotechnology, Santa Cruz, CA, United States) were added to each group before the start of the aggregation assay. For the ATP release experiments, the platelets were incubated with 50 ng/mL luciferin and D-luciferase (Cat No. 395; Chrono-Log Corp.) for 2 min before the addition of activators. The platelet aggregation and ATP release curves were traced during experiments.

### 2.4 Platelet Ca^2+^ and ROS release assays

The assay was performed as previously reported with minor modifications (Arthur et al., 2012; Ye et al., 2020). Briefly, WPs (500 × 10^9^/L) were labeled with 5 μM Fluo-3AM (Cat No. IF0150; Solarbio) or 10 μM DCFH-DA (Cat No. D6883; Sigma, St. Louis, MO, United States) at 37°C for 30 min protected from light. The platelets were then incubated with luteolin 2.5, 10, or 25 μM, BAY61-3606 (63 nM, Cat No.HY-76474; MCE), BAY61-3606+luteolin 25 μM, or an equal volume of DMSO at 37°C for 10 min. In addition, 1 mM CaCl_2_ and collagen or convulxin were added to start the reaction. Ca^2+^ release and ROS production were analyzed using a Fluoroskan Ascent fluorometer (Thermo Fisher, Waltham, Massachusetts, US) at excitation (ex)/emission (em) = 490/530 nm, and the curves were plotted in real-time.

WPs (500 × 10^9^/L) were incubated with convulxin 5 ng/mL and with the vehicle, luteolin 25 μM, BAY61-3606, or BAY61-3606+luteolin. A ROS probe via a fluorescent enzyme marker was added. O_2_^−^ and H_2_O_2_ were measured using 2 μM Amplex Red (Cat No. ST010; Beyotime Institute of Biotechnology, Shanghai, China) or 2 μM Dihydroethidium (DHE, Cat No. S0063; Beyotime Institute of Biotechnology, Shanghai, China). O_2_^−^ and H_2_O_2_ were analyzed using a Fluoroskan Ascent fluorometer (Thermo Fisher, Waltham, Massachusetts, United States).

### 2.5 Platelet LDH release assay

The effect of luteolin on platelet LDH release was assayed using the LDH cytotoxicity assay kit (Beyotime Institute of Biotechnology, Shanghai, China; C0017). WPs were incubated with 1% Triton X-100 solution (positive control), luteolin 2.5, 25, 100, or 200 μM, or an equal volume of DMSO at 37°C for 1 h. After incubation, the WPs were centrifuged at 2,000 × *g* for 10 min, and the supernatant was retained. The supernatant was processed according to the instructions, and the absorbance values were detected at 490 nm using a spectrophotometer Multiskan FC (ThermoFisher Scientific Inc., Waltham, MA, United States).

### 2.6 Platelet membrane protein expression

Platelet membrane protein expression experiments were performed as previously reported, with slight modifications (Babur et al., 2020). WPs were added with 10 μM indomethacin to prevent platelet aggregation. After incubation, the WPs were activated by adding 1 mM calcium chloride and agonists to platelets at 37°C. After 30 min, 10 μL of WPs was taken, and the reaction was terminated by adding 90 μL Tyrode’s buffer and 10 mM EDTA. PerCP-coupled anti-CD61 antibody (1:50; Cat No. 336410; Biolegend, San Diego, CA, United States), Alexa Fluor^®^ 647 labeled PAC-1 (1:50; Cat No. 362806; BioLegend), and FITC-coupled anti-CD62p antibody (1:50; Cat No. 555523; BD Biosciences, San Diego, CA, United States) were incubated with platelets for 30 min at 37°C in the dark. The platelet surface fluorescence intensity was analyzed using a FacsCanto II flow cytometer (BD Biosciences, San Diego, CA, United States) to analyze the expression of platelet surface membrane proteins. Cell membrane protein expression data were analyzed using FlowJo software V10.

### 2.7 Malonaldehyde (MDA), superoxide dismutase (SOD), glutathione peroxidase (GPx), and nicotinamide adenine dinucleotide phosphate (NADPH) oxidase activity assay

MDA, SOD, GPx, and NADPH oxidase activity were measured as previously described (Krotz et al., 2002; Paul et al., 2019; Pan et al., 2021). After incubation and activation, the reaction was stopped by adding 10 mM EGTA. The platelets were pelleted by centrifugation at 20,000 × *g* for 10 min at 4°C and resuspended in Tyrode’s buffer with 0.1% phosphatase and protease inhibitors (Cat No. 04906837001, Roche Applied Science, Penzberg, Germany; Cat No. P-8340, Sigma, St. Louis, MO, United States). The platelets were disrupted by passing them 10 times through a 29-gauge needle, followed by centrifugation at 2000 × *g* for 10 min at 4°C to separate the supernatant and precipitate. The detection of MDA, SOD, and GPx in the supernatant was performed according to the kit’s instructions for SOD (Cat No. BC0170, Solarbio Life Science, Beijing, China), MDA (Cat No. S0131S, Beyotime Institute of Biotechnology, Shanghai, China) and GPx (Cat No. S0056, Beyotime Institute of Biotechnology, Shanghai, China). In order to determine NADPH oxidase (NOX) activity, platelet lysate (12.5 μg) was incubated with 10 µM lucigenin and 0.5 mM NADPH at 37°C in the dark. The absorbance values were detected at 450 nm using a spectrophotometer Multiskan FC (ThermoFisher Scientific Inc., Waltham, MA, United States). Protein content was determined by the Bradford method.

### 2.8 Platelet-collagen static adhesion and platelet-fibrinogen spreading assays

As previously reported (Banfi et al., 2022), 50 μg/mL collagen and 25 μg/mL fibrinogen (Cat No. F3879-100 MG; Sigma-Aldrich, St. Louis, MO, United States) were used to coat coverslips overnight at 4°C. WPs were adjusted to 50 × 10^9^/L and incubated with different concentrations of luteolin (2.5, 10, and 25 μM) or DMSO for 10 min. The coated slides were washed three times with PBS and blocked with 1% BSA (prepared in PBS) for 1 h at room temperature. The blocking solution was removed, and WPs were added for adhesion experiments. The WPs were allowed to adhere to the slides for 15–60 min at 37°C. The unadhered platelets were removed by PBS. The platelets were fixed with 2% paraformaldehyde solution for 15 min, broken using 1% Triton-100 for 5 min, and stained with phalloidin (1:1,000, Cat No.17466-45-4, Sigma-Aldrich) for 30 min away from light at room temperature. The platelet morphology was observed using an Olympus ix73 microscope (Olympus Corp., Shinjuku, Japan) and photographed. The number of platelet adhesions and spreading area were analyzed and calculated using ImageJ software.

### 2.9 Clot retraction experiments

Clot retraction experiments were completed using the previously reported method (Zhang et al., 2015). Briefly, WPs were adjusted at 1,000 × 10^9^/L and incubated with different concentrations of luteolin (25 and 100 μM) or DMSO for 10 min. Then, 200 μL of WPs were placed in a siliconized glass tube with 1 mM CaCl_2_, 0.1 U/mL thrombin, and 10 µL PPP, mixed well, and left at 37°C. Pictures were taken every 5 min. The clot size was calculated using ImageJ software.

### 2.10 Ferric chloride-induced mesenteric thrombosis in mice

A method reported in the literature was used to observe mesenteric artery thrombosis in mice (Bonnard and Hagemeyer, 2015). The C57BL/6 male mice were divided into the DMSO, luteolin (35 μM/kg), and aspirin (0.5 mM/kg) groups (10 mice/group). The mice were anesthetized by an intraperitoneal injection of pentobarbital (45 mg/kg). They were given an intraperitoneal injection of DMSO, luteolin, or aspirin. They were also given an orbital vein injection of 5 μg/kg rhodamine (Cat No. 83697-250 MG; Sigma-Aldrich, St. Louis, MO, United States) (Bonnard and Hagemeyer, 2015), respectively. The mesenteric vessels were exposed by opening the abdominal cavity, and small mesenteric arteries with a diameter of approximately 150–250 μm were selected under the microscope. After injection of luteolin, DMSO, or aspirin for 30 min, a filter paper (approximately 5 mm long and 2 mm wide) soaked with 7.5% ferric chloride solution was placed over the target vessel for 2 min to induce vascular injury and thrombosis. During thrombosis, images were taken every 1 min for the first 10 min and every 5 min after 10 min using an MSHOT MD50 camera (Micro Shot Technology Ltd., Guangzhou, China) and an Olympus ix73 microscope (Olympus Corp., Shinjuku, Japan). The mice were observed for 60 min and sacrificed by the cervical dislocation method. The size of the thrombus formation was calculated using ImageJ software.

### 2.11 Collagen-epinephrine-induced acute pulmonary embolism model and oxidative markers in plasma

The acute pulmonary embolism model in mice was similar to the previously reported literature with slight modifications (Pan et al., 2021). The C57BL/6 male mice were divided into the DMSO and luteolin (35 μM/kg) groups and injected 30 min before modeling. Five hours after a retro-orbital sinus injection of collagen (100 μg/kg)-epinephrine (300 μg/kg), the mice were anesthetized by pentobarbital (45 mg/kg, i.p.). Whole blood obtained from the eyes was anticoagulated with EDTA·2Na. Whole blood was centrifuged at 3,000 rpm for 10 min to get plasma. Measurement of MDA and SOD in plasma followed the manufacturer’s protocols. Mice were sacrificed by cervical dislocation. The lung tissue was taken and fixed in 4% paraformaldehyde overnight.

### 2.12 Tail bleeding in mice

The tail break hemorrhage experiment in mice was performed as previously reported, with slight modifications (Liu et al., 2012). Anesthesia was performed by an intraperitoneal injection of pentobarbital (45 mg/kg), followed by an intraperitoneal injection of DMSO or luteolin, or aspirin. Before the experiment, the mouse tails were immersed in saline for 3 min at 37°C. Then, 30 min after injection, the tails were quickly cut off using a surgical knife blade and placed in saline at 37°C. The cutting site was 5 mm from the end of the tail tip. The bleeding time of the broken tails was observed and recorded.

### 2.13 Assessment of coagulation function in mice

The mice were anesthetized by an intraperitoneal injection of pentobarbital (45 mg/kg) and injected with DMSO or luteolin. Then, 30 min after administration, blood was removed from the eyes and anticoagulated with 3.8% sodium citrate. The plasma was obtained by centrifugation at 3,000 rpm for 10 min at room temperature, and coagulation-related indexes were measured using an STA Compact Max (Diagnostica Stago, Inc. Asnières-sur-Seine, France).

### 2.14 Western blot

In order to claim that luteolin inhibits GPVI signaling, signaling studies were performed under non-aggregating conditions (presence of 20 μg/mL integrin) and with inhibitors of secondary mediators (2 U/mL apyrase and 10 μM indomethacin), as previously reported (Babur et al., 2020). A lysis buffer (5x, PBS, PH7.4, comprising 2.5% sodium dodecyl sulfate [SDS], 5% deoxycholate, 5% vol/vol Triton X-100, phosphatase and proteinase inhibitors cocktails) was used to lyse the cells on ice for 30 min. The platelet lysate was centrifuged at 12,000 rpm for 10 min at 4°C. The supernatant was added to 5× protein loading buffer and boiled. The proteins were separated by 10% SDS-PAGE gels and transferred to membranes. The PVDF membranes were blocked in EveryBlot Blocking buffer (Cat No. 12010020; Bio-Rad, Hercules, CA, United States) for 10 min. Then, 1:1,000 primary antibodies were incubated overnight at 4°C on a shaker, including antibodies against Src (Cat No.2110), phospho-Src (Tyr416, Cat No.6943), phospho-Akt (Thr308, Cat No.2965), Akt (Cat No.4691), Syk (Cat No.12358), phospho-Syk (Tyr525/526, Cat No.2710), GSK-3β (Cat No.12456), phospho-GSK-3β (Ser9, Cat No.5558), PLCγ2 (Cat No.3872), phospho-SAPK/JNK (Thr183/Tyr185, Cat No.9251), SAPK/JNK (Cat No.9252), p38 MAPK (Cat No.8690), phospho -p38 MAPK (Thr180/Tyr182, Cat No.4511), phospho-p44/42 Erk 1/2 (Thr202/Tyr204, Cat No.4370), p44/42 Erk 1/2 (Cat No.4695), phospho-FAK (Tyr397, Cat No.8556), FAK (Cat No.3285) (all from Cell Signaling Technology Inc., Danvers, MA, United States), phospho-PLCγ2 (Tyr 759, Cat No. GTX133463) (GeneTex, Irvine, California, US), and *β*-actin (Cat No. GTX124214; GeneTex). The membranes were washed three times with TBST and incubated for 3 h at room temperature with rabbit (A7016, Beyotime Institute of Biotechnology, Shanghai, China) or mouse (A7028, Beyotime Institute of Biotechnology, Shanghai, China) secondary antibody (1:5,000). The bands were visualized using an ECL Substrate (Cat No. 1705062; Bio-Rad, Hercules, CA, United States) and a Tanon-5200 Chemiluminescent Imaging System (Tanon Science & Technology, Shanghai, China). The intensity of the target bands was assessed by ImageJ software.

### 2.15 Molecular docking

The docking experiments were performed as previously reported (Sang et al., 2020). The crystal structure of GPVI was obtained from the Protein Data Bank (PDB entry code: 2GI7). The luteolin structure was obtained from the PDB Database. Any heteroatoms and water molecules were removed for molecular docking studies. Semi-flexible docking was used to analyze the interaction of GPVI and luteolin by AutoDockToll 1.5.7. The receptor grid was generated using co-crystallization of GPO-3 repetition peptides with the GPVI D1 and D2 ectodomains (PDB files: 5OU8). The size of the affinity map was set at 92 × 92 × 62 Å, and spacing between the grid points was set to 0.375 Å. The view of the GPVI-LUT complex was generated using PyMOL (http://www.pymol.org/).

### 2.16 Surface plasmon resonance spectroscopy (SPR)

The SPR experiment was performed on a Biacore S200 instrument (GE Healthcare; Uppsala, Sweden) at 25°C. Recombinant human GPVI protein, rhGPVI (Cat. HY-P70180; MedChemExpress), was immobilized at the level of 4,400 response units, with 10 mM sodium acetate buffer (pH 4.5) on a Series S CM5 Sensor Chip (Cat. 29104988; cytiva) at the concentration of 50 μg/mL. Luteolin was diluted with PBS-P buffer supplemented with 5% DMSO to concentrations ranging from 0.78 to 12.5 μM. Different concentrations of luteolin were perfused through the channels at 30 μL/min for 90 s, and the response units were measured. The value of luteolin’s kinetic constants (*K*_d_, *K*_a_, and *K*_D_) to GPVI was analyzed using Biacore S200 Evaluation Software Version 1.1 (GE Healthcare) based on a 1:1 binding model.

### 2.17 Solid phase binding assays

rhGPVI was expressed and purified according to the method reported in the literature (Horii et al., 2006). rhGPVI was cross-linked with 10 μg/mL of cross-linking antibody His Tag Horseradish Peroxidase-conjugated Antibody (Cat. MAB050H; R&D Systems, Minneapolis, MN, United States). Solid phase assays were performed based on previously described procedures (Billiald et al., 2023). Collagen (5 μg/mL prepared in PBS) was used to coat 96-well plates overnight at 4°C. Non-specific sites were closed by Superblock (Cat. 37580; Thermo Fisher Scientific, Waltham, MA, United States). PBS-solubilized Luteolin (0–25 μM) and cross-linked GPVI (30 nM) were mixed in a 1:1 volume ratio and reacted with collagen-coated 96-well plates overnight at 4°C. 1-StepTM Ultra TMB-ELISA (Cat. 34022; Thermo Fisher Scientific, Waltham, MA, United States) was developed, and the reaction was stopped with a 2 M sulphuric acid solution. Absorbance values were read at 450 nm. At least three washes were performed with PBS between each of the above steps. Each spot was measured three times. The results were expressed as a absorption value of the A450 nm vs luteolin concentration, the inhibition curve was obtained by nonlinear fitting, and IC_50_ calculations were performed using GraphPad Prism 9.

### 2.18 Luteolin *in vivo* toxicity experiments

The C57BL/6 male mice were divided into the DMSO and luteolin (35 μM/kg) groups and injected every day. Blood was collected from the orbits every 3 days for blood tests. On the second day, after 15 days of intraperitoneal administration, pentobarbital (45 mg/kg) was injected intraperitoneally for anesthesia, and blood was sampled from the eyes. Whole blood was left for 30–60 min, and the supernatant was centrifuged at 3,000 rpm for serum liver and kidney function testing using Unicel DxC600 (Beckman Coulter, Inc. Brea, California, US). Finally, the mice were sacrificed by cervical dislocation, and the sternum was fixed in 4% paraformaldehyde.

### 2.19 Immunohistochemistry

Lung and sternum tissues were fixed in 4% paraformaldehyde, embedded in paraffin, and sectioned into 5-μm sections. After dewaxing and washing, the slices were incubated overnight at 4°C for immunostaining with anti-mouse CD41 antibody (Cat No. GTX113758, GeneTex Inc., Irvine, CA, United States) 0.63 μg/mL or with anti-mouse CD42b antibody (Cat No. 12860-1-AP; Proteintech Group Inc., Chicago, IL, United States) 1 μg/mL or an equivalent concentration of polyclonal non-immune IgG (Cat No. GTX213110-01; GeneTex) as control. The sections were washed three times and incubated for 50 min at room temperature with secondary antibodies. The nuclei were counterstained using hematoxylin. The tissues were observed using 3,3′-Diaminobenzidine tetrahydrochloride.

### 2.20 Statistical analysis

All data were expressed as mean ± standard error of the mean (SEM) and analyzed using GraphPad Prism 9 (GraphPad Software Inc., San Diego, CA, United States). Unless otherwise stated, unpaired t-tests were used to compare the differences between the two groups. ANOVA followed by Dunnett’s test was applied to multiple comparisons. *p*-values <0.05 were considered statistically significant.

## 3 Results

### 3.1 Luteolin selectively inhibits GPVI-mediated platelet aggregation and ATP release

**FIGURE 1:**
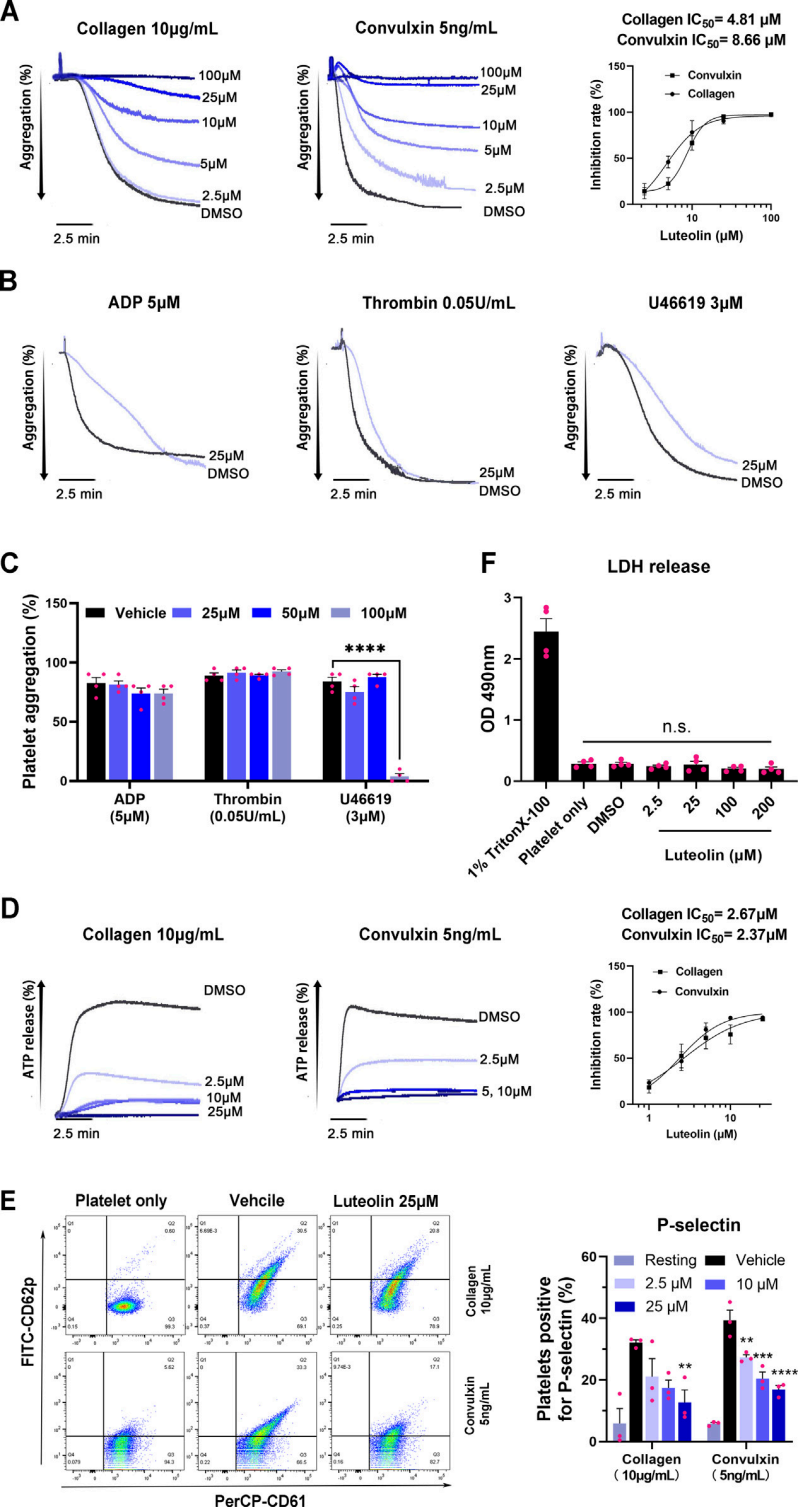
Effect of luteolin on collagen- and convulxin-induced platelet aggregation, ATP release, P-selectin expression, and LDH release. Human washed platelets (WPs) were incubated with different concentrations of luteolin or DMSO at 37°C for 10 min after the addition of indicated agonists. **(A–D)** After incubation, the platelet aggregation and ATP release curves were depicted in real-time, and the inhibition rate curves were drawn (A, far right) to calculate the IC_50_ values using nonlinear regression. **(B)** Platelet aggregation curves under different stimulations. **(C)** The platelet aggregation rates were calculated from the curves in **(B)**. **(D)** ATP release and inhibition rate IC_50_ values using nonlinear regression **(E)** Flow cytometric scatter plots were performed for CD62p and CD61. **(F)** Platelets were incubated at 37°C for 60 min with different doses of luteolin, DMSO, or Triton X-100. The LDH release was measured. All experimental results are expressed as mean ± SEM (n ≥ 3). Compared with the Vehicle(DMSO) group, **p < 0.01, ***p < 0.001, ****p < 0.0001. Data were analyzed with one-way ANOVA followed by Dunnett’s test.

Studies reported that luteolin significantly inhibits platelet aggregation and ATP release mediated by low doses of collagen, ADP, and U46619 (Choi et al., 2015; Lu et al., 2021), but luteolin’s dose and incubation time are different. In this study, we re-screened the effect of luteolin on platelet aggregation and ATP release induced by various activators. As shown in Figure 1A, luteolin significantly inhibited platelet aggregation induced by collagen (10 μg/mL) and GPVI-specific activator convulxin (5 ng/mL) with IC_50_ of 4.81 and 8.66 μM, respectively. The inhibition ratio of 25 μM of luteolin under collagen and GPVI stimulation was approximately 95%. Luteolin (25 μM) had no significant effect on platelet aggregation induced by ADP (5 μM), U46619 (3 μM), and thrombin (0.05 U/mL). Luteolin (100 μM) significantly inhibited platelet aggregation induced by U46619 (Figures 1B, C). The results suggest that luteolin mainly inhibited GPVI-mediated platelet aggregation under the experimental conditions of this study.

The initial platelet activation is accompanied by the release of ATP and P-selectin, and the release response is earlier and more sensitive to platelet activation than the aggregation response (Hvas, 2023). Therefore, we investigated the effect of luteolin on collagen- and GPVI-mediated platelet release of ATP and P-selectin. Similar to the aggregation results, luteolin significantly inhibited convulxin- and collagen-induced platelet ATP (Figure 1D) and P-selectin (Figure 1E) in a concentration-dependent manner. Luteolin had little effect on LDH release (Figure 1F). The results suggest that luteolin significantly inhibits platelet release responses induced by collagen and convulxin.

### 3.2 Luteolin inhibits GPVI-mediated platelet superoxide production, promotes SOD and GPx activity and impairs NOXs activity

**FIGURE 2:**
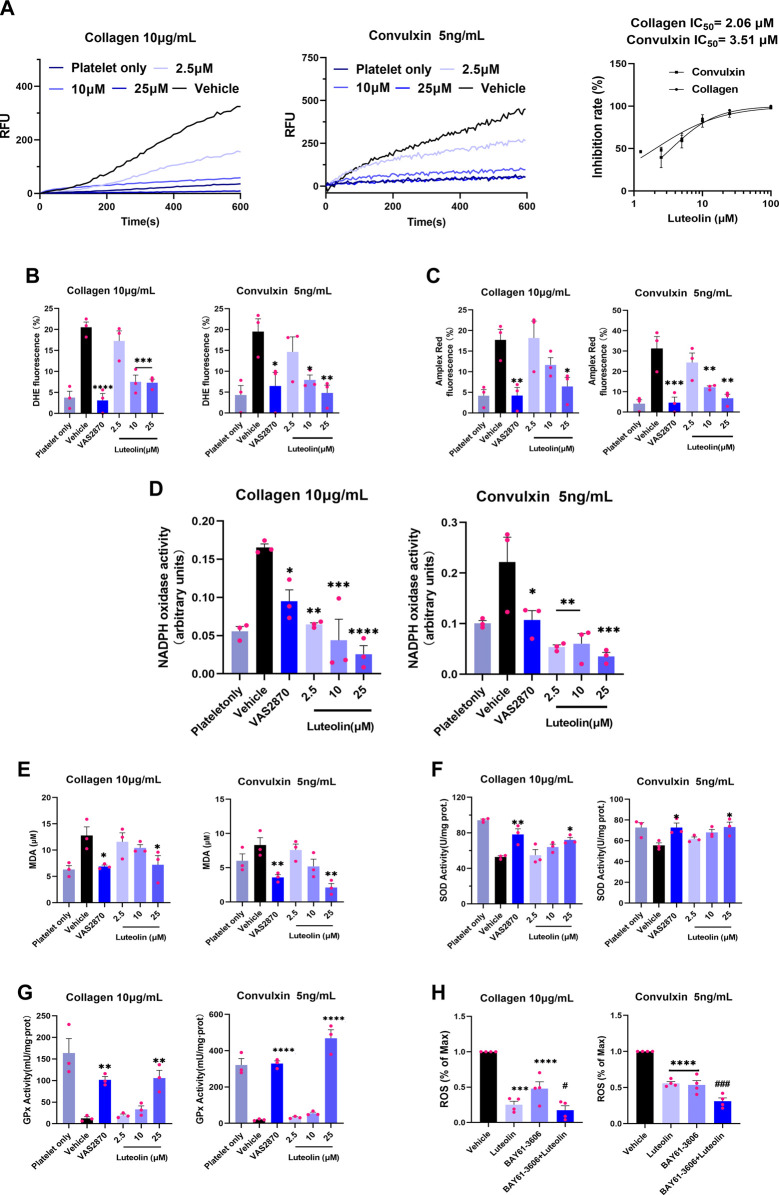
Effect of luteolin on GPVI-mediated oxidative stress in human platelets. The reactive oxygen species (ROS) assay probes DCFH-DA (5 μM), 2 μM Amplex Red, and 2 μM DHE were used to pre-stained human washed platelets (WPs). The WPs were incubated with different concentrations of luteolin, DMSO, and Syk-specific inhibitor (BAY61-3606) for 10 min at 37°C. **(A)** Collagen (10 μg/mL) and GPVI-specific activator convulxin (5 ng/mL) activation of human platelets. Human platelet ROS release was monitored in real-time at ex/em = 480/530 nm. RFU: relative fluorescence value. **(B,C)** Flow cytometry detection of platelet superoxide anion and H_2_O_2_ release. **(D)** Effect of luteolin and VAS2870 (10 μM) on collagen (10 μg/mL) and convulxin (5 ng/mL) -induced NOXs activity. **(E–G)** Platelet lysate supernatant MDA, SOD, and GPx activities were measured. **(H)** Effect of luteolin (25 μM) and BAY61-3606 (63 nM) on collagen- (10 μg/mL) and convulxin (5 ng/mL)-induced platelet ROS release. Absorbance values were detected at 450 nm after the reaction of platelet membrane protein lysate with NADPH (0.5 mM) and lucigenin (10 μM). Compared with the BAY61-3603 group, ^#^ *p* < 0.05, ^###^ *p* < 0.001. Data were analyzed with one-way ANOVA followed by Dunnett’s test. Values are expressed as mean ± standard error, n ≥ 3. Compared with the Vehicle (DMSO) group, *p < 0.05, **p < 0.01, ***p < 0.001, ****p < 0.0001.

Collagen-bound platelet GPVI induces activation of NOXs, and NOXs activation mediates intracellular ROS release (Qiao et al., 2018; Masselli et al., 2020; Morotti et al., 2022). The ROS generated by GPVI-induced activation of NOXs is the main source of collagen-induced platelet ROS production. Therefore, we first observed the effect of luteolin on collagen- and GPVI-mediated ROS release from human WPs using a ROS probe via a fluorescent enzyme marker. As shown in Figure 2A, luteolin inhibited collagen- and convulxin-induced ROS release from human platelets with efficiency in accordance with the inhibition of aggregation, with IC_50_ of 2.06 and 3.51 μM, respectively. Meanwhile, luteolin also inhibited platelet superoxide anion and hydrogen peroxide release (Figures 2B, C). Secondly, based on the principle that the catalytic substrate NADPH of NOXs produces superoxide anion, which induces lucigenin and releases energy, the activity of NOXs was measured by the absorbance value of the reduced luster sperm at OD of 450 nm by a spectrophotometer. As shown in Figure 2D, convulxin and collagen induced NOXs activation and degraded NADPH, and the absorbance value at 450 nm was increased compared with non-activated human platelets, and NOXs inhibitor (VAS2870) and luteolin significantly decreased the convulxin- and collagen induced NOXs activity. Luteolin also inhibited the convulxin- and collagen-induced MDA activity and promoted SOD, and GPx’s activity (Figures 2E–G).

Platelet ROS release is mainly mediated through Syk-dependent and Syk-independent signaling (Arthur et al., 2012; Canobbio et al., 2015). A Syk-specific inhibitor, BAY61-3606, was used to explore the effect of luteolin on the signaling pathway of GPVI activation-mediated platelet ROS release. Luteolin or BAY61-3606 alone decreased convulxin-induced platelet ROS release, and the combination of luteolin and BAY61-3606 produced a stronger ability to inhibit platelet ROS release than that produced by BAY61-3606 alone; a similar result was found for collagen-induced platelet ROS release with luteolin or BAY61-3606 alone decreasing levels and the decrease being greater in combination (Figure 2H). Therefore, we hypothesized that luteolin could inhibit GPVI-mediated platelet ROS release either through Syk-dependent or Syk-independent signaling. The findings suggest that luteolin inhibits GPVI-mediated platelet oxidative stress. Luteolin inhibits NOXs activation and ROS release, and this effect might be related to the inhibition of Syk-dependent and Syk-independent signaling.

### 3.3 Luteolin inhibits platelet static adhesion to collagen

**FIGURE 3:**
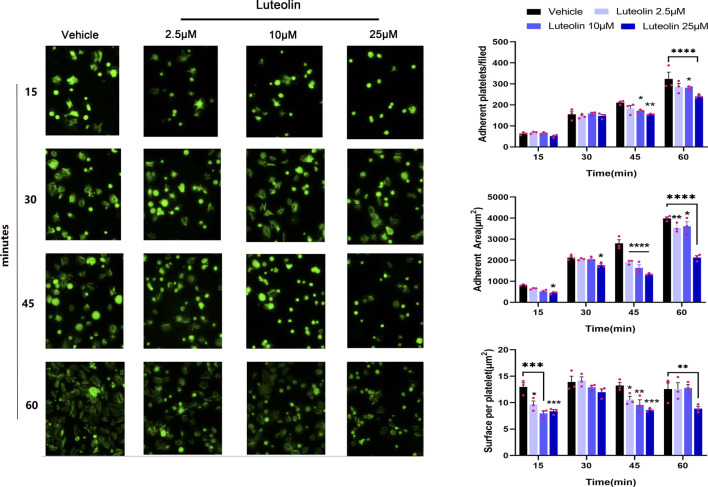
Effect of luteolin on platelet static adhesion to the collagen surface. Adhesion of human washed platelets (WPs) on the surface of collagen (50 μg/mL) collagen-coated coverslips (n = 3) after incubation with different concentrations of luteolin or DMSO at 37°C for 10 min. Magnification ×40. The bar graphs compare the number and area of platelet adhesions. The experimental results are expressed as mean ± SEM, analyzed with two-way ANOVA followed by Dunnett’s test. Compared with the vehicle group, *p < 0.05, **p < 0.01, ***p < 0.001, ****p < 0.0001.

Exposure of the collagen matrix at the vascular injury and platelet adhesion to the collagen surface initiates thrombosis (Sun et al., 2022). This adhesion step mainly involves integrin a2β1 and GPVI. We investigated the effect of luteolin on platelet static adhesion to the collagen surface by immunofluorescence. As shown in Figure 3, the number of adherent platelets increased with time. Luteolin 10 and 25 µM significantly decreased platelet adherence at 45 and 60 min compared with the vehicle. Compared with the vehicle, luteolin 2.5 and 10 µM decreased the surface per platelet at 15 and 45 min, while luteolin 25 µM decreased the surface per platelet at 15, 45, and 60 min. The adherent area increased in all groups with time. Luteolin 25 µM decreased the adherent area at 15, 30, 45, and 60 min compared with the vehicle, while luteolin at 2.5 and 10 µM slightly decreased the adherent area at 60 min. Therefore, these results suggest that increasing luteolin concentration had a more obvious effect on cell adhesion.

### 3.4 Luteolin inhibits integrin αIIbβ3-mediated platelet activation

**FIGURE 4:**
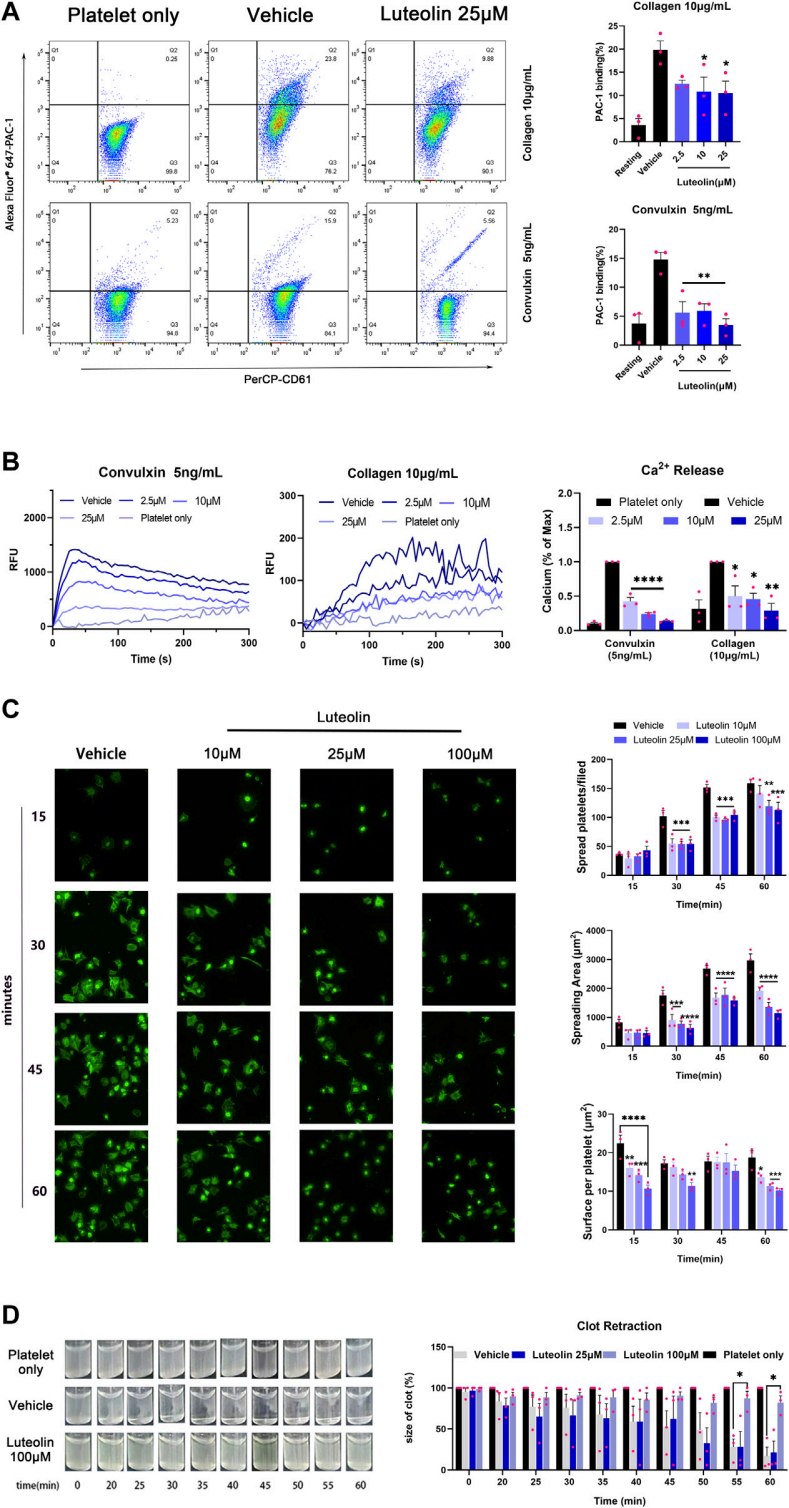
Effect of luteolin on integrin αIIbβ3-mediated “inside-out” and “outside-in” activation in human platelet. **(A)** Washed platelets (WPs) were incubated with DMSO or luteolin at 37°C for 10 min CaCl_2_ (1 mM) and collagen (10 μg/mL) or convulxin (5 ng/mL) were added to stimulate the WPs. The expression of the PAC-1 protein on the platelet surface was analyzed using flow cytometry. n = 3. Statistical analysis of PAC-1 cells was calculated by the FlowJo software. **(B)** Convulxin (5 ng/mL) and collagen (10 μg/mL) induced platelets intracellular calcium release were recorded and traced using a Fluorescence microplate reader at ex/em = 480/530 nm (left panel). n = 3. **(C)** The WPs were spread on 25 μg/mL of fibrinogen-coated slides Magnification ×40. n = 3. **(D)** WPs were incubated with 1 mM CaCl_2_ with 0.1 U/mL thrombin at 37°C and photographed every 5 min to observe clot retraction, n = 3. Data are expressed as mean ± SEM, analyzed with ANOVA followed by Dunnett’s test. Compared with the vehicle group, *p < 0.05, **p < 0.01, ***p < 0.001, ****p < 0.0001.

Integrin αIIbβ3-mediated platelet activation is the final common pathway for platelet activation induced by multiple activators (Joo, 2012). Previous findings suggest that luteolin inhibits ADP-induced expression of PAC-1, the activating form of platelet integrin αIIbβ3 (Luo et al., 2017). We investigated the effect of luteolin on integrin αIIbβ3-mediated platelet activation by immunofluorescence and flow cytometry. First, luteolin dose-dependently inhibited the collagen- and convulxin-induced platelets integrin αIIbβ3 “inside-out” activation, i.e., calcium release and PAC-1 expression (Figures 4A, B). Luteolin also inhibited integrin αIIbβ3-mediated platelet spreading on fibrinogen (Figure 4C). Luteolin (100 μM) inhibited thrombin-induced clot retraction (Figure 4D). The experiments demonstrated that luteolin significantly inhibited integrin αIIbβ3-mediated platelet activation.

### 3.5 Luteolin binds GPVI

**FIGURE 5:**
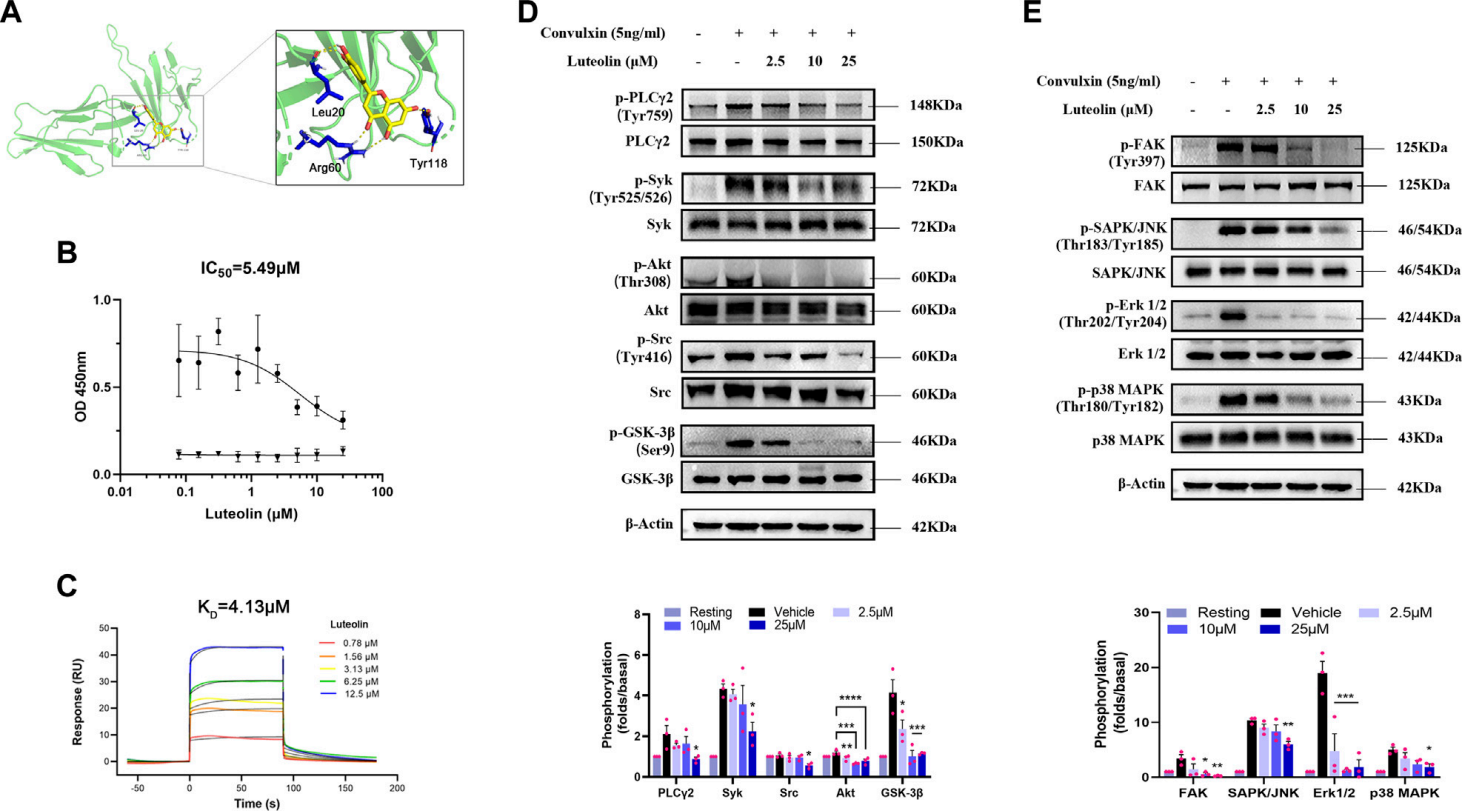
Binding of luteolin to GPVI and effect on convulxin-induced activation of ITAM and MAPK signaling pathways. **(A)** Molecular docking showing the schematic diagram of luteolin binding to GPVI (PDB code: 2GI7). Yellow: luteolin; green: GPVI; blue: interacting amino acid residues; yellow dashed lines: hydrogen bonds. **(B)** Inhibition of rhGPVI binding to immobilized collagen by increasing concentrations of luteolin. The binding of rhGPVI at 30 nM (black circles) to collagen was dose-dependently inhibited by luteolin with IC_50_ values of 5.49 µM using nonlinear regression. In contrast, luteolin had no chromogenic reaction on the 5 μg/mL (black triangles) collagen. n = 3. **(C)** SPR analysis of the interaction between luteolin and GPVI. **(D, E)** Effect of luteolin on convulxin-induced activation of platelet ITAM and MAPK signaling pathway molecule. n = 3. Results are expressed as mean ± SEM analyzed with one-way ANOVA followed by Dunnett’s test. Compared with the vehicle group, *p < 0.05, **p < 0.01, ***p < 0.001, ****p < 0.0001.

We used molecular docking to explore the spatial binding sites of the luteolin-GPVI interaction. Luteolin can form hydrogen bonds with GPVI residues Leu20, Arg60, and Tyr118 (Figure 5A). Consistent with our findings, a point mutation in multiple amino acid sites Arg60, Gly30, Trp76, Glu40, Val34, Leu36, Arg38, Lys59, and Arg166 affects the binding of CRP and collagen to GPVI (Lecut et al., 2004; Brondijk et al., 2010; Feitsma et al., 2022). Solid-binding assays were also used to investigate the effect of luteolin on the binding of GPVI to collagen (Figure 5B). Luteolin dose-dependently inhibited the binding of rhGPVI to collagen with an IC_50_ of 5.49 μM. The above experiments suggest that the interaction between luteolin and GPVI may have influenced the binding of GPVI to collagen. The above results showed that luteolin had a significant inhibitory effect on platelet activation induced by GPVI-specific activators. In order to clarify the interaction between luteolin and GPVI, we used SPR and explored the direct interaction between luteolin and GPVI. Figure 5C shows the real-time curve of the interaction between luteolin and GPVI. By calculation, the *K*_D_ of the interaction between luteolin and GPVI was 4.13 μM, *K*_a_ was 7,227 m^−1^s^−1^, and *K*_d_ was 0.03 s^−1^.

### 3.6 Effect of luteolin on GPVI-mediated intracellular ITAM and MAPK signaling

The above results indicated that luteolin significantly inhibited platelet aggregation, release, and adhesion induced by collagen and convulxin and that luteolin binds to GPVI. GPVI transduces intracellular activation signals through Fcγ receptors. FAK activation promotes phosphorylation and activation of Src family-activated kinases (SFKs) (Guidetti et al., 2019). SFKs activate immunoreceptor-activated motifs in the cytoplasmic region of the immunoreceptor tyrosine-based activation motif (ITAM) activation and recruitment and phosphorylation of splenic tyrosine kinase (Syk). Activated Syk leads to PI3K-Akt pathway and PLCγ2 activation. As expected, luteolin inhibited the phosphorylation of the ITAM signaling pathway molecules Src, Syk, PLCγ2, and Akt and also inhibited the phosphorylation of GSK-3β, a molecule downstream of PI3K-Akt pathway signaling, suggesting that the inhibitory effect of luteolin is related to both ITAM and Akt (Figure 5D). Platelet MAPK signaling molecules mainly include ERKs (extracellular-signal-regulated kinases), JNKs (Jun amino-terminal kinases), and p38/SAPKs (stress-activated protein kinases). GPVI also can activate the MAPK signaling pathway (Patel and Naik, 2020). Therefore, we investigated the effect of convulxin on the phosphorylation of Erk 1/2, SAPK/JNK, and p38 MAPK, the major signaling molecules of MAPK (Figure 5E). Luteolin had a significant inhibitory effect on convulxin-induced phosphorylation of MAPK and ITAM.

### 3.7 Luteolin inhibits thrombus formation *in vivo*

**FIGURE 6:**
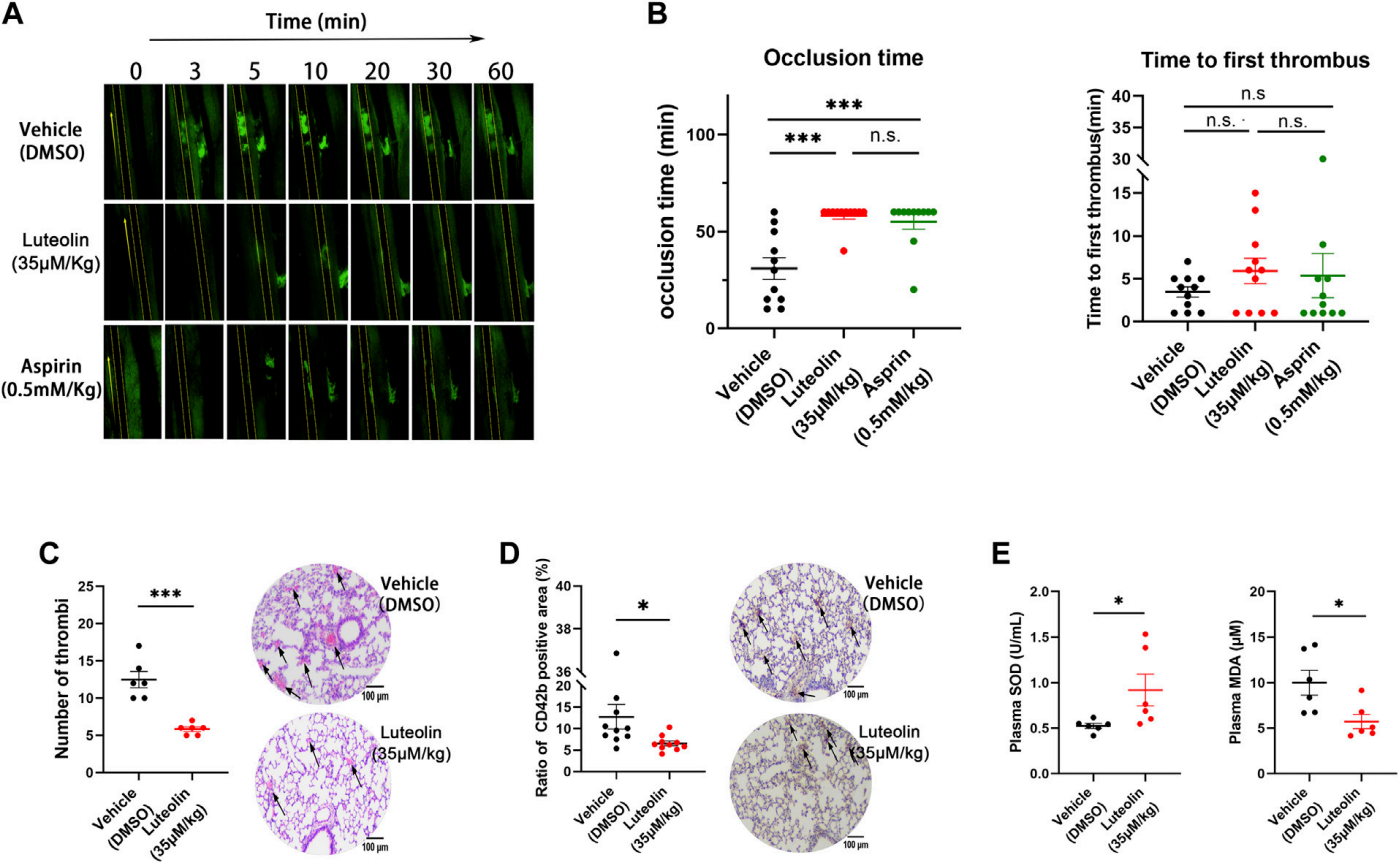
Effect of luteolin on thrombus formation and oxidative stress *in vivo*. **(A,B)** Effect of luteolin on ferric chloride-induced thrombosis in the mesenteric arteries of mice. Magnification ×4. n = 10. **(C,D)** Effects of luteolin on collagen and epinephrine injection induced pulmonary thrombosis in mice. H&E **(C)** and CD41 **(D)** immunohistochemical analyses were performed, and the photo-grams were magnified at ×10, with arrows indicating thrombus (n = 6). **(E)** Effect of luteolin suppresses on malonaldehyde (MDA) generation and superoxide dismutase (SOD) activity in plasma of pulmonary embolism mice (n = 6). Results are expressed as mean ± SEM. Differences between DMSO and luteolin groups were analyzed with unpaired t-tests to compare the differences between the two groups. One-way ANOVA followed by Dunnett’s test was applied to multiple comparisons. *p < 0.05, ***p < 0.001.

Arterial thrombosis was examined in a mouse model of mesenteric thrombosis induced by ferric chloride (Bonnard and Hagemeyer, 2015). The *in vivo* experiments were used to evaluate the effect of luteolin on the platelet function. The results showed that the 35 μM/kg luteolin group and the 0.5 mM/kg aspirin group could significantly prolong the time of clot obstruction of blood vessels (Figure 6B). Neither the 0.5 mM/kg aspirin group or 35 μM/kg luteolin group prolonged the time of initial thrombus formation (Figure 6A, B).

We used the intravenous collagen and epinephrine model to explore the effects of luteolin on pulmonary thrombosis *in vivo*. As shown in Figures 6C, D, collagen-epinephrine injection resulted in significant thrombosis in mouse lungs, and luteolin significantly attenuated the area and size of thrombus formation. Moreover, consistent with the role of oxidative stress in pulmonary embolism, we found that the administration of luteolin significantly decreased MDA levels and increased SOD activity in plasma of mice with pulmonary embolism (Figure 6E). The antioxidant role of luteolin in mice with pulmonary embolism is in line with its inhibition of ROS production from human platelets stimulated with collagen and convulxin (Figure 2).

### 3.8 Luteolin led to no significant effect on hemostasis and coagulation function in mice

**FIGURE 7:**
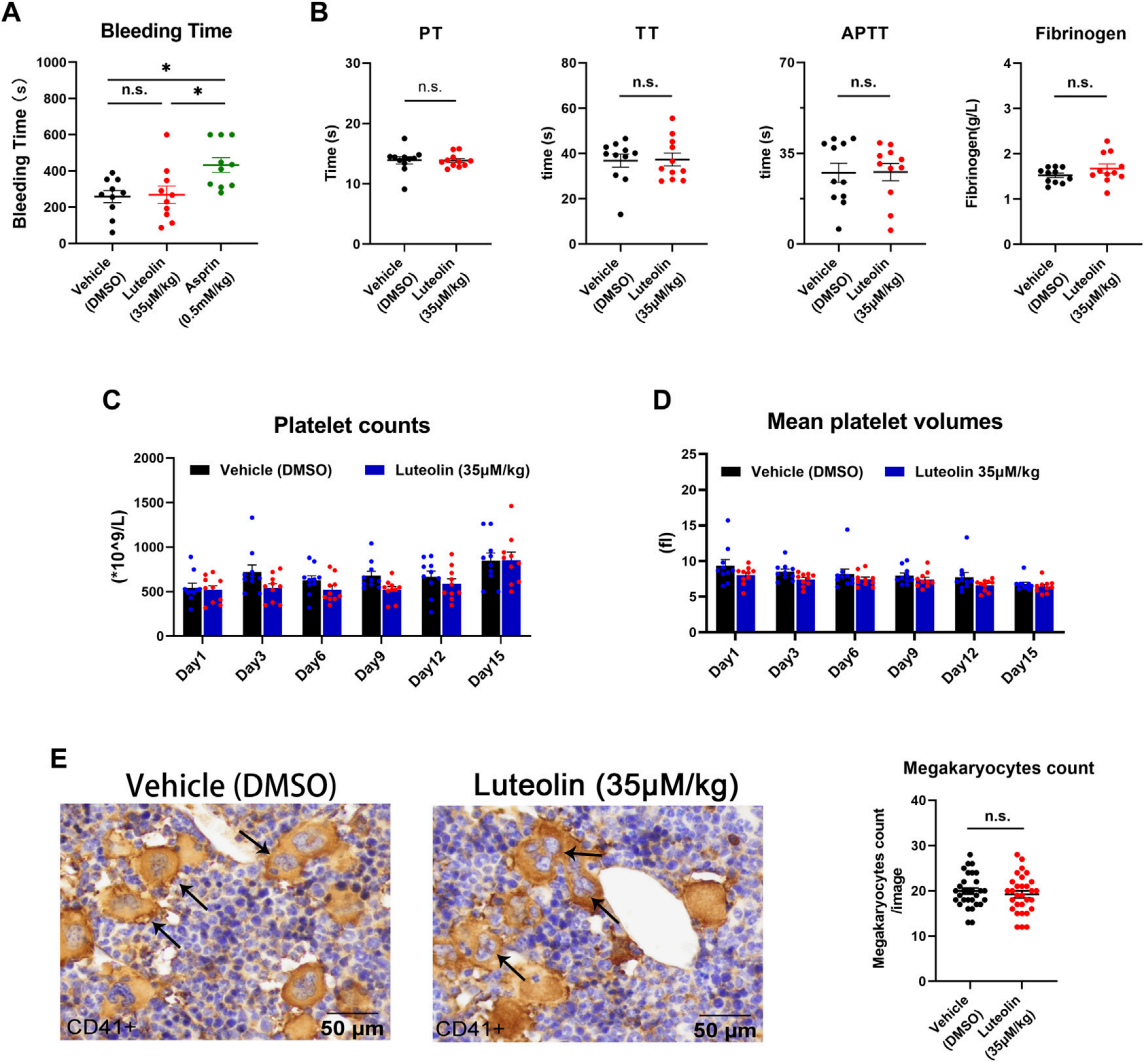
Effect of luteolin on hemostasis, coagulation, and platelet production in mice. **(A)** Mice were anesthetized 30 min after injection of DMSO or luteolin and aspirin. Effect of luteolin on tail bleeding time in mice. n = 10. **(B)** Coagulation analysis, n = 10. **(C,D)** Luteolin was injected intraperitoneally daily for 15 days. Changes in platelet count and volume in mice during administration, n = 10. **(E)** Immunohistochemistry of sternum bone marrow sections from luteolin-treated (35 μM/kg/day) or DMSO-treated mice. Mouse sternum was taken and prepared for immunohistochemical analysis. CD41 staining was positive for macrophages, as shown by the arrow. The photo-grams were magnified at ×40. The bar graph shows the numbers of megakaryocytes from six fields from 10 mice in each group. Results are shown as mean ± SEM, Differences between DMSO, aspirin, and luteolin groups were analyzed with unpaired t-tests to compare the differences between the two groups. One-way ANOVA followed by Dunnett’s test was applied to multiple comparisons. *p < 0.05.

The above experiments verified the significant inhibitory effect of luteolin on platelet activation and thrombosis formation. Now, we observed the effect of luteolin on physiological hemostasis and coagulation function in mice. The tail bleeding time was significantly prolonged in the positive control aspirin group, while 35 μM/kg luteolin had no significant prolonging effect on tail bleeding in mice compared with the DMSO control group, indicating that luteolin may not affect physiological hemostasis in mice under the present experimental conditions (Figure 7A). Meanwhile, we found that luteolin also had no significant effects on these four coagulation indexes when we tested prothrombin time (PT), activated partial thromboplastin time (APTT), thrombin time (TT), and the plasma concentration of fibrinogen in mice (Figure 7B). Therefore, we conclude from the results that luteolin does not significantly affect physiological hemostasis and coagulation function.

### 3.9 Intraperitoneal injection of luteolin has no significant effect on platelet production

The above findings suggest that luteolin binds GPVI and inhibits platelet function. Platelets are functional fragments shed by megakaryocytes, and it is unclear whether luteolin affects platelet production. In order to assess the effect of luteolin on platelet production *in vivo*, we first compared the number of megakaryocytes (CD41 [+]) in immunohistochemically stained sections of bone marrow from mice treated with luteolin (35 μM/kg) or an equivalent volume of DMSO for 15 days.

There were no significant differences in the peripheral platelet count and mean platelet volume between the luteolin and DMSO groups (Figures 7C, D). There were no significant differences in the megakaryocyte counts in bone marrow between the luteolin and DMSO groups (Figure 7E). The blood cell counts were normal and had no obvious changes between the two groups (Table 1). The results suggest that under the present experimental conditions, luteolin has no significant effect on blood routine and platelet production in C57BL/6 mice *in vivo*.

**TABLE 1:** Effects of luteolin on the biochemical and hematological parameters in mice plasma.

Variable	Vehicle (DMSO)	Luteolin (35 μM/kg)	*p*
White blood cells (×10^9^/L)	1.32 ± 0.12	1.35 ± 0.10	0.8500
Lymphocytes (×10^9^/L)	0.74 ± 0.05	0.80 ± 0.04	0.4124
Monocytes (×10^9^/L)	0.24 ± 0.04	0.25 ± 0.04	0.8880
Granulocytes (×10^9^/L)	0.39 ± 0.07	0.36 ± 0.03	0.6503
Lymphocyte %	55.31 ± 3.42	57.39 ± 1.59	0.5890
Monocyte %	17.20 ± 1.79	17.39 ± 1.56	0.9375
Granulocyte %	27.49 ± 2.41	25.22 ± 0.74	0.3811
Hemoglobin (g/L)	12.98 ± 0.52	13.02 ± 0.63	0.9553
Red blood cells (×10^12^/L)	0.60 ± 0.04	0.65 ± 0.03	0.2658
Total bilirubin (µmol/L)	5.04 ± 0.25	5.24 ± 0.49	0.7199
Aspartate aminotransferase (U/L)	133.20 ± 4.88	147.20 ± 7.03	0.1185
Alanine aminotransferase (U/L)	55.98 ± 2.16	66.21 ± 5.35	0.0931
Alkaline phosphatase (U/L)	67.50 ± 5.64	75.00 ± 7.34	0.4284
γ-glutamyl transferase (U/L)	5.10 ± 0.72	6.20 ± 1.24	0.4523
Blood urea nitrogen (mmol/L)	9.96 ± 0.54	10.14 ± 0.40	0.7911
Creatinine (µmol/L)	16.52 ± 1.20	17.22 ± 2.61	0.8102

### 3.10 Intraperitoneal injection of luteolin had no significant toxic effects in C57BL/6 mice

Based on the LD_50_ of intraperitoneal luteolin injection, the dose used in this experiment was safe (Aziz et al., 2018). Still, the toxic effects of short-term luteolin administration on platelets and C57BL/6 mice are unclear. Therefore, we first investigated the effect of luteolin on platelet LDH release *in vitro*. Luteolin below 200 μM did not significantly increase platelet LDH release within 1 h compared with DMSO treatment (Figure 1F). As shown in Table 1, the liver and kidney functions were normal after luteolin (35 μM/kg) administration for 15 days, indicating that luteolin had no significant toxic effects in C57BL/6 mice.

## 4 Discussion

In this study, we focused on the effects of luteolin on GPVI-mediated platelet activation, thrombosis, and platelet production. First, we verified the antiplatelet and antithrombotic effects of luteolin, and the results support the antiplatelet activity of luteolin to the GPVI receptor. Molecular docking simulation suggested the formation of hydrogen bonds between luteolin and GPVI. The effect of luteolin on the binding of GPVI to collagen was also demonstrated by solid-binding experiments. This study is also the first to report the direct interaction between luteolin and GPVI by SPR. The significant inhibition of GPVI-mediated platelet activation and thrombosis by luteolin was verified by *in vitro* and *in vivo* experiments in human platelets and mice. Secondly, we reported for the first time that luteolin has a weak effect on coagulation, hemostasis, and platelet production in mice. Therefore, the results suggest anti-GPVI is an effective and safe antiplatelet treatment strategy. The results provide a reference for the search and design of anti-GPVI small molecule drugs.

However, it is important to note that while numerous studies have suggested that GPVI is a safe and efficient target for antithrombotic therapy, and GPVI antibodies with high blocking efficiency have been developed, they still have the disadvantages of high cost, low bioavailability of oral formulations, inappropriate storage and transport, and immune response. Therefore, the development of small molecule GPVI inhibitors with low cost, high efficiency and safety, and easy storage and transport is still a key direction for antiplatelet drug development. Currently, few small molecule inhibitors of GPVI have been reported, and both GPVI and previous drug candidates have been unsuccessful in the clinic, resulting in the development and application of small molecule inhibitors (Damaskinaki et al., 2021).

The main reasons and challenges for the difficulties in the development and application of small molecule inhibitors are: 1, the interaction between protein ligands and receptors is complex and involves multiple sites and large areas of interaction. Whereas small molecules essentially bind to key residues in the receptor and interact over small areas, this complexity makes it difficult to perform simulations in small molecules; 2, many protein-protein interactions (PPIs) are relatively flat at the site, lack a well-defined binding pocket, and exist at multiple contacts, as is the case for GPVI, which also leads to the identification of small-molecule inhibitors to become challenging; 3, as GPVI does not have a high affinity ligand, it is difficult to study and design small molecule inhibitors based on the ligand.

Studies with Glenzocimab and nanomolar-level nanobody 2 have shown that GPVI signaling requires an active structural domain-exchange conformation of GPVI and provide a novel mechanism for GPVI activation, one that binds to the structural domain inducing a spatial site-blocking and structural modification that inhibits the interaction of GPVI with its primary ligands, independent of the primary ligands (Slater et al., 2021; Billiald et al., 2023). The new binding site can provide a new research direction for small molecule inhibitors - inhibition of structural domains - from another level. Therefore, in order to develop small molecule drugs, it is a very challenging task to predict the binding sites of small molecules and GPVI and explore the mechanism because different sites exert the same or similar inhibitory effects.

Platelet activation is an intricate and complex process involving many feedback loops and interactions between different pathways. Such complexity aims to provide active and adequate protection against bleeding while avoiding excessive and life-threatening thrombosis (Bye et al., 2016; Estevez and Du, 2017). In particular, platelets rely on endogenous secondary signal amplification mechanisms and their regulation to achieve continuous aggregation and thrombosis. Activated platelets secrete adenosine diphosphate (ADP), serotonin, and thromboxane A2 (T_x_A_2_), which are not only necessary for platelet activation by low doses of collagen, but also reactivate platelets and activate the integrin αIIbβ3 via an “inside-out” signaling pathway (Bye et al., 2016; Estevez and Du, 2017). Studies from platelet aggregation and secretion reported that luteolin could bind directly to the T_X_A_2_ receptor and directly inhibit platelet activation (Guerrero et al., 2005). When the arachidonic acid (AA) metabolic pathway is inhibited by flavonoids, it indirectly affects T_X_A_2_ production in platelets, so platelet activation pathways closely related to T_X_A_2_ secretion, such as P2Y1 and P2Y12-mediated ADP-induced platelet calcium, cAMP, AA, and T_X_A_2_ production, are significantly inhibited (Lu et al., 2021). Our study found 25 μM luteolin had no significant effect on platelet aggregation induced by 3 μM U46619, but 100 μM luteolin significantly inhibited platelet aggregation induced by U46619. This is in general agreement with the previous study (Guerrero et al., 2005)where luteolin inhibited 2 μM U46619 with an IC_50_ of 47.0 ± 13.3 μM (Guerrero et al., 2005). Slight differences between results should be expected with differences in buffers and instruments between studies. In this study, we also found 25 μM luteolin had no significant effect on platelet aggregation induced by 5 μM ADP. This might be considered to contrast with previous results where 25–100 μM luteolin inhibited ADP induced platelet aggregation (Lu et al., 2021). However, this can be explained by differences in experimental procedures between the studies, in particular the previous study used 1 h incubation times in contrast to the 10 min used in our study. Moreover, our experiments have also confirmed that high concentrations of luteolin (25–100 μM) affect the function of αIIbβ3, and once it affects the function of this receptor, it may also have an impact on platelet aggregation induced by various activators of platelets. Therefore, in overall agreement with the literature, in the present study, high doses of luteolin inhibited U46619-induced platelet aggregation effect. In addition, luteolin significantly inhibited integrin αIIbβ3-mediated “outside-in” platelet activation, which might be due to the direct stimulation of GPVI by platelet integrin αIIbβ3 ligands fibrinogen and fibronectin, enhancing platelet activation (Jourdi et al., 2021). GPVI and receptors such as T_X_A_2_, P2Y1 and P2Y12 are necessary to maintain αIIbβ3-mediated “outside-in” platelet activation, and the effect of luteolin on these receptors might affect αIIbβ3-mediated “outside-in” platelet activation. Platelet αIIbβ3 receptor-mediated platelet activation is the final common pathway of receptor-mediated platelet activation, including GPVI, T_X_A_2_, P2Y1 and P2Y12, and recent studies have demonstrated that fibrinogen and fibronectin can also activate GPVI, which in turn enhances platelet activation and thrombosis (Li et al., 2010; Alshehri et al., 2015; Onselaer et al., 2017; Induruwa et al., 2018). Thus, the function of luteolin in inhibiting platelet activation is primarily related to GPVI, and luteolin may also be able to influence the transduction of multiple platelet activation signaling pathways via GPVI.

Increased platelet ROS is strongly associated with thrombophilia, hypertension, diabetes, hypercholesterolemia, and metabolic syndrome (Masselli et al., 2020; Zhang et al., 2023). Non-activated human platelets contain a certain amount of intracellular ROS. Platelet activation leads to increased release of intracellular ROS, and GPVI mediates the main source of intracellular ROS production (Pignatelli et al., 1998; Arthur et al., 2012). The generated ROS can also regulate platelet adhesion and activation (Begonja et al., 2005; Jang et al., 2014). Several studies also reported that antioxidants inhibit platelet activity and thrombosis (Zhang et al., 2021; Gu et al., 2022). The present study showed that luteolin inhibits the GPVI-mediated release of platelet ROS *in vitro*. Regarding intracellular signaling studies, this study revealed that luteolin significantly inhibited the activation of MAPK signaling pathway molecules. ROS can act as a second messenger to activate the MAPK signaling pathway and is likewise under the control of the MAPK signaling pathway, as previously reported (Begonja et al., 2005). Therefore, luteolin has significant inhibitory effects on anti-oxidative stress *in vitro*, as supported by previous studies (Nazari et al., 2013; Ou et al., 2019; Ntalouka and Tsirivakou, 2023).

Luteolin gavage for 7 days at 20 mg/kg/day can significantly reduce thrombosis and infarct area in rats (Liu et al., 2022). In addition, 200 mg/kg/d luteolin significantly reduced the low-density lipoprotein content in diet-induced obese mice (Cheurfa et al., 2019). Previous studies showed that luteolin is mainly metabolized by the liver and excreted by the kidneys (Hodek et al., 2002; Chen et al., 2014). In the present study, the concentration of luteolin used in the *in vitro* experiments was similar to the dose used in a previous study (Choi et al., 2015), and the *in vivo* experiments used the safe dose of luteolin, i.e., 1/18 the LD_50_ (180 mg/kg) (Aziz et al., 2018). The results also suggested that after 15 days of continuous intraperitoneal injection of luteolin, the liver and kidney function indices of the mice were not significantly affected compared to the DMSO control group and were within the normal range. Since there is still little information on the effects of luteolin on platelets and the hematological system, we investigated the effects of luteolin on platelet production and monitored the changes in the blood routine of the mice during administration, but there were no differences in the hematological parameters. The results also revealed that luteolin weakly affected hemostasis and the coagulation system. These experimental results further suggest that luteolin could be expected to be developed as a safe antiplatelet drug.

We used an SPR approach to study the interaction of GPVI with luteolin, and the results suggested a direct interaction between luteolin and GPVI. We also used a molecular docking approach to explore the spatial structure analysis of luteolin with GPVI. Although luteolin did not bind to the binding pocket of the GPVI-specific ligand CPR, its binding site was close to the pocket of the active site (Lecut et al., 2004; Horii et al., 2006; Feitsma et al., 2022). Our solid phase binding assay also demonstrated that luteolin could affect the binding of GPVI to collagen. These findings further confirm that luteolin binds GPVI and inhibits platelet GPVI-mediated platelet activation.

This study had limitations. Although the interaction and spatial binding sites of luteolin with GPVI are currently understood using SPR and docking, we still lack crystal data on the interaction of GPVI with luteolin. Further analysis of the crystal structure of the complex will help us to understand the interaction sites with GPVI with flavonoids. In addition, mutant proteins should be generated using point mutation of amino acids at the binding site to confirm the binding site between luteolin and GPVI using either a solid-binding assay or an SPR assay. Furthermore, combined with previous reports suggesting that the inhibitory effect of luteolin on platelet activation is multi-targeted and multi-effective, although we confirmed *in vivo* studies in mice that luteolin has a weak effect on platelet production, hemostasis, and coagulation system, human data still need to be analyzed in further clinical studies. Transcriptomics analyses could be performed to examine the pathways affected by luteolin.

In conclusion, luteolin is an inhibitor of the platelet GPVI receptor and has no significant effect on the hematologic system, including coagulation and platelet production *in vivo* while exerting antiplatelet and antithrombotic effects. Combined with the antioxidant stress-protective effect of luteolin, luteolin is expected to be a safe and effective anti-atherosclerotic and thrombogenic drug.

## Data Availability

The original contributions presented in the study are included in the article/Supplementary Material, further inquiries can be directed to the corresponding author.
