# Supplementary figures and images for: Luteolin inhibits GPVI-mediated platelet activation, oxidative stress, and thrombosis
Source: Front Pharmacol. 2023 Oct 31;14:1255069. doi: 10.3389/fphar.2023.1255069 (PMC10644720; doi:10.3389/fphar.2023.1255069)

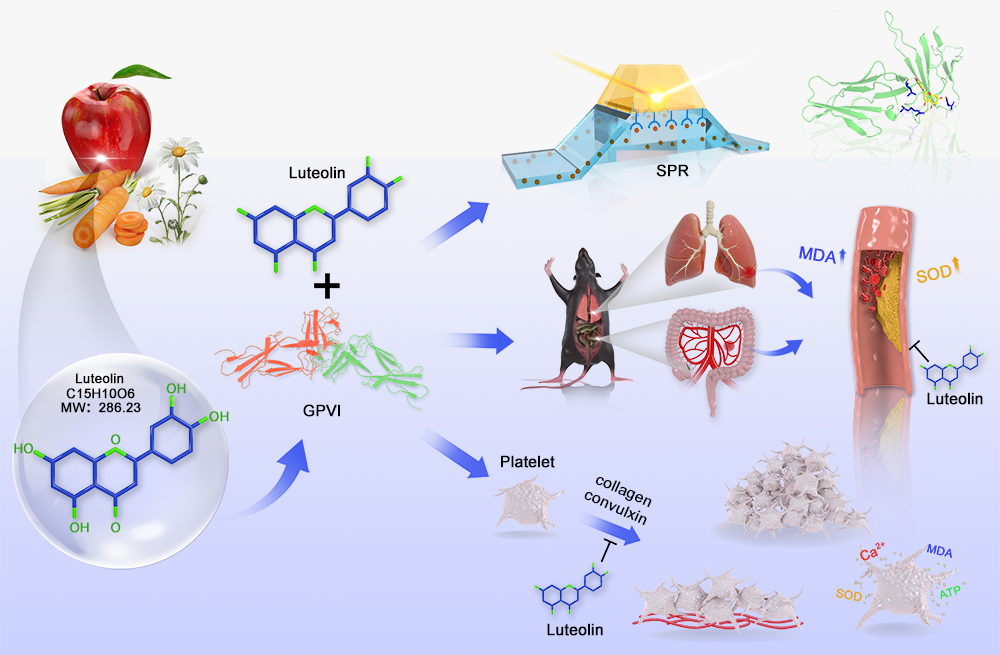

Supplement: Supplementary file 1 [file Image1.TIF]
